# The Key Role of Non-governmental Organizations (NGOs) Activities in Viral Hepatitis Elimination Programs

**DOI:** 10.15171/ijhpm.2018.125

**Published:** 2018-12-22

**Authors:** Farzaneh Padami, Seyed Moayed Alavian, Monir Niazi

**Affiliations:** ^1^Baqiyatallah Research Center for Gastroenterology and Liver Diseases (BRCGL), Baqiyatallah University of Medical Sciences, Tehran, Iran.; ^2^Middle East Liver Diseases (MELD) Center, Tehran, Iran

## Dear Editor,

Over the last decades, one of the most important health‏ problems which has affected the health of millions of people annually and has led to their morbidity and mortality has been viral hepatitis.‏ It can be said that hepatitis B virus (HBV) infection is the main cause of chronic liver disease in the world,‏ but thanks to the vaccinating and the screening of pregnant women, its burden has been decreased dramatically during the last decade.^1,2^ Furthermore, the receiving of infected blood and blood products was the most common transmission route for hepatitis C virus (HCV) in the world until 1992. Fortunately, with implementation of mandatory HCV screening of blood and blood products, the number of post-transfusion infections has been decreased dramatically in special patients,^1^ but injection drug misuse (IDM) has remained the main risk factor for acquiring of HCV infection in our community.

Since one of the main causes of viral hepatitis is related to lack of awareness about the routes through which these diseases can be transmitted,^3^ the effective role of non-governmental organizations (NGOs) like World Hepatitis Alliance in raising public awareness about viral hepatitis, changing policy and taking action to find those unaware of their diseases is undeniable. Therefore, in line with the global movement to eliminate HCV by the year 2030, Iran Hepatitis Network as an official member of World Hepatitis Alliance tried its best to pilot a NGO under the name of *Hope Health Club*, as an educational model, to raise public awareness about health issues in general and liver diseases in particular. As a matter of fact, this NGO aims to transfer its experiences and achievements to the other communities by providing a general framework for training the community.

The vision of Hope Health Club is to eliminate hepatitis C and control hepatitis B by the year 2030 in Iran through reinforcing education programs by the focus on self-care culture, and enjoying the capacity of organs, charities and public associations in the field of health. This NGO’s mission is both raising public awareness about medical and psychological effects of viral hepatitis on the life of patients and society. As a matter of fact, this NGO is planned to activate all organs to educate and motivate people to take precautionary actions during next five years.

In order to reach this goal, *Hope Health Club* with the cooperation of other organisms and NGOs has attempted to hold public conferences by the name of “*Hope.*” It is notable that thanks to the people’s warm reception, 12 “*Hope*” conferences have been held in different cities up to now‏ and more than one thousand people participated in them. These conferences emphasize on adopting a healthy lifestyle by avoiding risky sexual behaviors, IDM, insanitary tattooing and bloodletting, and so forth.

Besides face to face training, several instructional video clips and more than 40 books and brochures in the field of liver diseases have been provided by Hope Health Club’s website which are easily available for download.^4^ These books and brochures have been translated into 9 different languages and have been downloaded more than 20 000 times by Iranians and non-Iranians. Moreover, an instructional brochure was designed to raise children awareness about liver’s functions and viral hepatitis and distributed in some primary schools.

In addition to diverse activities on the website, *Hope Health Club* plays an important role in social networks such as Instagram, Telegram, and a mobile application with more than 30 000 subscribers. All these try to raise public awareness by making friendly connection and an opportunity for their subscribers to ask specialists their questions. Furthermore, holding attractive contests such as drawing and illustration, photography, and book reading with the theme of hepatitis elimination has been considered as another successful and undeniable step in attracting community’s attention to this global movement.^5^

Finally, among this foundation’s activities, the election of 15 Health Ambassadors among prominent faces like actors, directors, doctors, athletes and those who care about health issue is noteworthy. These ambassadors attempt to motivate people to avoid risky behaviors which endanger their health.

Hope Health Club as a non-profit foundation functioning independently of government is financially supported by philanthropic organizations and companies interested in advertising in website and mobile application.

Therefore, it is suggested that the implementation of such training framework could have a positive effect in control and elimination of viral hepatitis.

## Ethical issues

Not applicable.

## Competing interests

Authors declare that they have no competing interests.

## Authors’ contributions

FP drafted the manuscript and and other authors read and edited the draft manuscript.

## Authors’ affiliations

^1^Baqiyatallah Research Center for Gastroenterology and Liver Diseases (BRCGL), Baqiyatallah University of Medical Sciences, Tehran, Iran. ^2^Middle East Liver Diseases (MELD) Center, Tehran, Iran.
